# *N*^6^-Methyladenosine (m^6^A)-Circular RNA Pappalysin 1 (circPAPPA) from Cashmere Goats: Identification, Regulatory Network and Expression Potentially Regulated by Methylation in Secondary Hair Follicles Within the First Intron of Its Host Gene

**DOI:** 10.3390/ani15040581

**Published:** 2025-02-18

**Authors:** Man Bai, Jincheng Shen, Yixing Fan, Ruqing Xu, Taiyu Hui, Yubo Zhu, Qi Zhang, Jialiang Zhang, Zeying Wang, Wenlin Bai

**Affiliations:** 1 College of Animal Science & Veterinary Medicine, Shenyang Agricultural University, Shenyang 110866, China; 2 Engineering Research Center for Animal Molecular Genetics and Breeding of Liaoning Province, Shenyang 110866, China

**Keywords:** cashmere goats, circPAPPA, *N*^6^-methyladenosine, first intron methylation, regulatory network, expression pattern

## Abstract

*N*^6^-methyladenosine (m^6^A) is one of the most abundant modifications in eukaryotic RNA molecules and mediates the functional exertion of RNA molecules. We characterized the circPAPPA and validated its potential m^6^A modification sites in secondary hair follicles (SHFs) of cashmere goats. Furthermore, we generated integrated regulatory networks of the circPAPPA along with enrichment analysis of signaling pathways. We also explored the potential relationship of circPAPPA expression with the first intron methylation of the *PAPPA* gene in SHFs of cashmere goats. Host source analysis revealed that circPAPPA is derived from the complete exon 2 of the PAPPA gene, spliced in reverse orientation, and predominantly localized in the cytoplasm of SHF stem cells in cashmere goats. The circPAPPA was verified to contain at least four m^6^A modification sites in SHFs of cashmere goats, including m^6^A-450/456, m^6^A-852, m^6^A-900, and m^6^A-963. The generated regulatory network indicated complex and diverse regulatory relationships of m^6^A-circPAPPA with its putative regulatory molecules, including miRNAs, mRNAs, and proteins. Enrichment analysis of signaling pathways showed that m^6^A-circPAPPA might play multiple functional roles in the growth and development of SHF in cashmere goats through the putative regulatory network mediated by its target miRNAs and regulatory proteins. The first intron methylation of the *PAPPA* gene most likely is significantly involved in the dynamic expression of m^6^A-circPAPPA in cashmere goat SHFs. Results from this study provided novel information to elucidate the biological roles and functional regulatory pathways of m^6^A-circPAPPA in SHF development and the growth of cashmere goat fiber.

## Simple Summary

Cashmere goats are an important livestock species in the agricultural and pastoral areas of northern China. Cashmere is one of the main products of cashmere goats. It is derived from secondary hair follicles (SHFs) in the skin tissue of cashmere goats. However, the precise molecular mechanism of the growth of cashmere fibers is still not fully understood. In this study, we characterized the *N*^6^-methyladenosine (m^6^A)-modified circPAPPA and confirmed that circPAPPA contained at least four m^6^A modification sites in the SHFs of cashmere goats, including m^6^A-450/456, m^6^A-852, m^6^A-900, and m^6^A-963. The m^6^A-modified circPAPPA exhibits higher expression in the cytoplasm of SHF stem cells of cashmere goats in comparison to the nucleus. Based on bioinformatics analysis, m^6^A-modified circPAPPA may play multiple functional roles in SHF development and the growth of cashmere through potential regulatory networks mediated by miRNAs and RNA-binding proteins. First, intron methylation of the host gene (*PAPPA*) of m^6^A-modified circPAPPA is most likely significantly involved in the dynamic expression of m^6^A-modified circPAPPA in the SHFs of cashmere goats.

## 1. Introduction

Cashmere goats are an important livestock species in the agricultural and pastoral areas of northern China, with great economic significance for local agro-pastoralists [1]. As is well known, cashmere, a kind of natural protein fiber, is one of the main products of cashmere goats. As a high-grade textile raw material, cashmere is noted for its unique fiber properties, including softness, lightness, shine, and warmth retention. It is derived from the dynamic mini-organs, named secondary hair follicles (SHFs), in the skin tissue of cashmere goats. The morphogenesis and growth of cashmere fibers are closely regulated by seasonal SHF activity with three main stages: anagen, catagen, and telogen [2]. In previous studies, it has been demonstrated that many endogenous regulatory molecules are implicated in the growth regulation of cashmere fibers through highly coordinated interactions among functional genes [3], non-coding RNAs [4], and signaling pathways [5]. However, the precise molecular mechanism of cashmere growth is still not fully understood. Therefore, it is crucial to identify and characterize novel regulatory factors that may be significantly involved in the growth regulation of cashmere fibers in cashmere goats.

*N*^6^-methyladenosine (m^6^A) is one of the most abundant modifications in eukaryotic linear RNAs, including mRNAs and long non-coding RNAs [6]. The m^6^A modifications essentially mediate the functional exertion of RNA molecules [7]. Over the past few years, interestingly, extensive m^6^A modifications were also identified in circular RNA (circRNA) molecules among various species with significant functional roles [8,9,10]. It was demonstrated that m^6^A modification of circCCDC134 mediated by ALKBH5 promoted cervical cancer metastasis through heightening IF1A expression [11]. CircMDK was found to facilitate tumorigenesis via its m^6^A-mediated upregulation and acts as a nanotherapeutic target in hepatocellular carcinoma [8]. The m^6^A modifications of circRNAs also play essential roles in pork quality [10] and the inflammation process of bovine mammary epithelial cells injured by *Staphylococcus aureus* and *Escherichia coli* [12]. There was also evidence that m^6^A modification mediated the function of circRNA-08436 in the lipid metabolism of dairy goat mammary glands through the miR-195/ELOVL6 axis [13]. However, m^6^A-modified circRNAs have been less extensively investigated in cashmere goats. Several m^6^A-modified circRNAs have potential functional roles in the anagen SHFs of cashmere goats [14]. For example, circZNF638 and m^6^A-circERCC6 facilitate induced activation of SHF stem cells in a m^6^A-dependent manner through the miR-361-5p/Wnt5a axis and miR-412-3p/BNC2 axis, respectively [15,16].

CircPAPPA was initially identified as chi_circ_2829 from the skin tissue of cashmere goats, with *PAPPA* being defined as its host gene, and it exhibited significantly higher expression at anagen SHFs in comparison to telogen SHFs [17]. However, there is no further information available regarding circPAPPA with its transcription source, functionally possible regulation pathways, and expression regulatory mechanism in cashmere SHFs. The PAPPA has been identified as a proteolytic enzyme that is able to cleave IGFBPs, thereby increasing IGF bioactivity and, hence, promoting IGF signaling [18] as an essential regulatory system for hair follicle development [19]. Therefore, we speculate that circPAPPA plays a crucial role in SHF development and cashmere fiber growth in goats. This is the first time the molecular characterization of circPAPPA was analyzed, and its potential m^6^A modification sites were validated. Integrated regulatory networks of circPAPPA were generated, along with enrichment analysis of signaling pathways based on bioinformatics tools. We further explored the potential regulatory mechanism of circPAPPA expression in the SHFs of cashmere goats. The results obtained in this investigation contribute to elucidating the biological roles and functional regulatory pathways of circPAPPA in SHF development and the growth of cashmere fibers in goats.

## 2. Materials and Methods

### 2.1. Nucleic Acid Samples and Cell Culture

The study protocols were approved by the Experimental Animal Ethics and Welfare Committee of Shenyang Agricultural University (No. 2023030208), with the experiments performed based on the approved guidelines. In this study, total RNA and genomic DNA were utilized that were isolated from the SHFs of cashmere goats in our recent investigation [20]. For subcellular localization analysis of circPAPPA, SHF stem cells of cashmere goats were used that have been stored in our laboratory [15]. We cultured the cells in Dulbecco’s modified Eagle medium (DMEM)/F12, including 10% fetal bovine serum (Hyclone, Logan, UT, USA). The cells were incubated under a condition of 37 °C with CO_2_ concentration of 5%, and the medium was changed every 2 days. Total RNA was extracted with RNAiso reagent kit according to the manufacturer’s instructions (TaKaRa, Dalian, China). Cytoplasmic and nuclear RNA were extracted from collected SHF stem cells of cashmere goats using Cytoplasmic and Nuclear RNA Purification Kits (AmyJet, Wuhan, China).

### 2.2. Host Source Analysis of m^6^A-circPAPPA in Cashmere Goats Along with Its Sequence Structural Features

CircPAPPA was previously identified as chi_circ_2829 from the skin tissue of cashmere goats, where *PAPPA* had been defined as its host gene [17]. To further define its precise transcription region within the PAPPA gene, alignment was performed by the linear sequence of circPAPPA against genome datasets of goats (*Capra hircus*) on the NCBI website (Genome assembly ARS1.2, https://www.ncbi.nlm.nih.gov/datasets/genome/GCF_001704415.2, last access: 25 September 2024). Potential m^6^A sites within the circPAPPA sequence were screened by the use of the SRAMP procedure (http://www.cuilab.cn/sramp, last access: 25 September 2024). Potential target miRNAs on circPAPPA were screened using the custom sub-procedure of the miRDB procedure (http://www.mirdb.org, last access: 25 September 2024), where human data were utilized owing to the unavailability of goat data in miRDB datasets. Unidentified miRNAs in goats were excluded from the target miRNAs using a combined miRNA database: miRNAsong (https://www2.med.muni.cz/histology/miRNAsong/index.php, last access: 25 September 2024). Also, the mature sequences of the goat miRNAs were retrieved from the miRNAsong website. The binding structural feature between each selected miRNA and circPAPPA was analyzed by an online service program, RNAhybrid (https://bibiserv.cebitec.uni-bielefeld.de/rnahybrid/, last access: 25 September 2024). The coding potential of circPAPPA was predicted by an online service program: RNAsamba (https://rnasamba.lge.ibi.unicamp.br/, last access: 25 September 2024). Subsequently, potential ORFs within circPAPPA were screened by an online service program, the ORF Finder at the NCBI website (https://www.ncbi.nlm.nih.gov/orffinder, last access: 25 September 2024).

### 2.3. Validation of m^6^A Modification Sites of circPAPPA with Its Subcellular Localization

Validation of m^6^A modification sites of circPAPPA was carried out by Me-RIP technique followed by qPCR analysis [21]. The isolated total RNA of 100 μg was digested by RNase R (Geneseed, Guangzhou, China). Subsequently, the RNA samples were further concentrated by the Monarch^®^ RNA Cleanup Kit (NEB, Ipswich, MA, USA). The fragmentation of the resulting RNA sample was performed by NEBNext^®^ Magnesium RNA Fragmentation Module (NEB, Ipswich, MA, USA). The fragmented RNA product of 2 μg was stored for usage as input control. We incubated the half-fragmented RNA sample with 2 μg of anti-m^6^A antibody (Synaptic Systems, Gottingen, Germany) or IgG (Cell Signaling Technology, Danvers, MA, USA) for 4 h at 4 °C. Dynabeads Protein A (Thermo Scientific, Rockford, IL, USA) was subjected to incubation with the complex of RNA and antibody for 2 h at 4 °C. Subsequently, RNA was isolated and the first strand cDNAs were synthesized with random primers. The relative abundance of each m^6^A modification site on circPAPPA was measured by qPCR technique, with the data being normalized to the input control [21].

Subcellular localization of circPAPPA expression was carried out based on the usage of SHF stem cells of cashmere goats that have been stored in our laboratory [15]. We measured the relative abundance of circPAPPA on both nuclear and cytoplasm RNA from SHF stem cells by qPCR analysis. The *snRNA-U6* and *GAPDH* were used as internal controls for the nuclear and cytoplasmic fractions of the analyzed SHF stem cells, respectively. The relative abundance of the circPAPPA was calculated by the 2^−ΔΔ*Ct*^ method.

### 2.4. Regulatory Network Construction of circPAPPA Along with Enrichment Analysis of Signaling Pathways

To generate the ceRNA regulatory network of circPAPPA, we predicted potential target genes of the miRNAs (potential targets of circPAPPA) using the custom sub-procedure of the miRDB program (http://www.mirdb.org, last access: 25 September 2024). The Cyotoscape (Version 2.8.3) program was used to construct and visualize the ceRNA network of circPAPPA [22]. Signaling pathway enrichment of circPAPPA potentially regulatory genes was performed by the CluePedia built-in plugin of the Cyotoscape program under default settings (http://www.ici.upmc.fr/cluepedia/, last access: 27 September 2024).

In generating the regulatory network of circPAPPA with its regulatory target proteins, we predicted direct interaction proteins (DIPs) of circPAPPA using the database of RNA-binding protein specificities, RBPDB (http://rbpdb.ccbr.utoronto.ca/, last access: 27 September 2024). The resulting regulatory relationships of circPAPPA with its target proteins were provided as a network that was further extended by the FunRich program (www.funrich.org, last access: 27 September 2024). Enrichment analysis of signaling pathways on the resulting target proteins was performed by the CluePedia built-in plugin of the Cyotoscape program under the default settings (http://www.ici.upmc.fr/cluepedia/, last access: 27 September 2024). Finally, we provided the results on significantly enriched signaling pathways as a chordmap that was generated by the SRplot procedure [23].

### 2.5. Expression Detection of circPAPPA Along with Methylation Analysis Within First Intron of Its Host Gene (PAPPA) in Cashmere Goat SHFs

For expression analysis of circPAPPA in cashmere goat SHFs (anagen, catagen, telogen), random primers (Sangon, Shanghai, China) were used for the reverse transcriptions on total RNA that was extracted in our recent investigation [20]. The qPCR analysis was carried out by divergent primers (Table 1) in a final volume of 25 μL. The reaction assay consisted of 2.0 μL of first-strand cDNA solution, 1.0 μL (10 μM) of each primer, 12.5 μL of Green Premix Ex Taq II TB (Tli RNaseH Plus, TaKaRa, Dalian, China) and 8.5 μL of ddH_2_O water. Forty amplification cycles comprised 95 °C for 5 s, 59 °C for 30 s, and 72 °C for 30 s. Relative expression of circPAPPA was calculated using the 2^−^^∆∆*Ct*^ method, where the expression of the housekeeping gene *GAPDH* was used as the internal control.

To test the first intron methylation of the circPAPPA host gene (PAPPA), we screened possible CpG islands within the 2000 bp region immediately downstream of exon 1 of the PAPPA gene based on the Methyl Primer Express procedure (Version 1.0, Applied Biosystems, Foster, CA, USA). We screened possible binding sites of transcription factors within the amplified first intron region of the *PAPPA* gene by the AliBaba procedure (Verison 2.1, http://gene-regulation.com/pub/programs/alibaba2/index.html, last access: 28 September 2024). We treated genomic DNA samples using MethylCode™ Bisulfite Conversion Kit (Invitrogen, Shanghai, China) and pooled them into three groups: anagen, catagen and telogen. Bisulfite sequencing PCR (BSP-PCR) amplification was carried out using BSP-primers of *PAPPA* (Table 1) under the assay described above. The amplified PCR product was purified using a DNA purification kit (TaKaRa, Dalian, China) and ligated into pMD18-T Vector (TaKaRa, Dalian, China) that was further propagated in competent *E. coli* DH5α cells. Ten positive clones were sequenced for telogen, anagen and catagen. The resulting methylation level was presented by the use of the QUMA program [24].

### 2.6. Statistical Analysis

Results were presented as mean ± standard error, and statistical analysis was carried out by SPSS 17.0 software (SPSS Inc., Chicago, IL, USA). Differences between groups were analyzed using the Student’s *t*-test with *p*-values less than 0.05 and 0.01, which were considered significant and highly significant, respectively. Ultimately, the results were presented with GraphPad Prism for Windows (Version 8.3.0, San Diego, CA, USA, www.graphpad.com, last access: 4 October 2024).

## 3. Results and Discussion

### 3.1. Host Source Analysis of circPAPPA in Cashmere Goats with Its Sequence Structural Features

**Figure 1:**
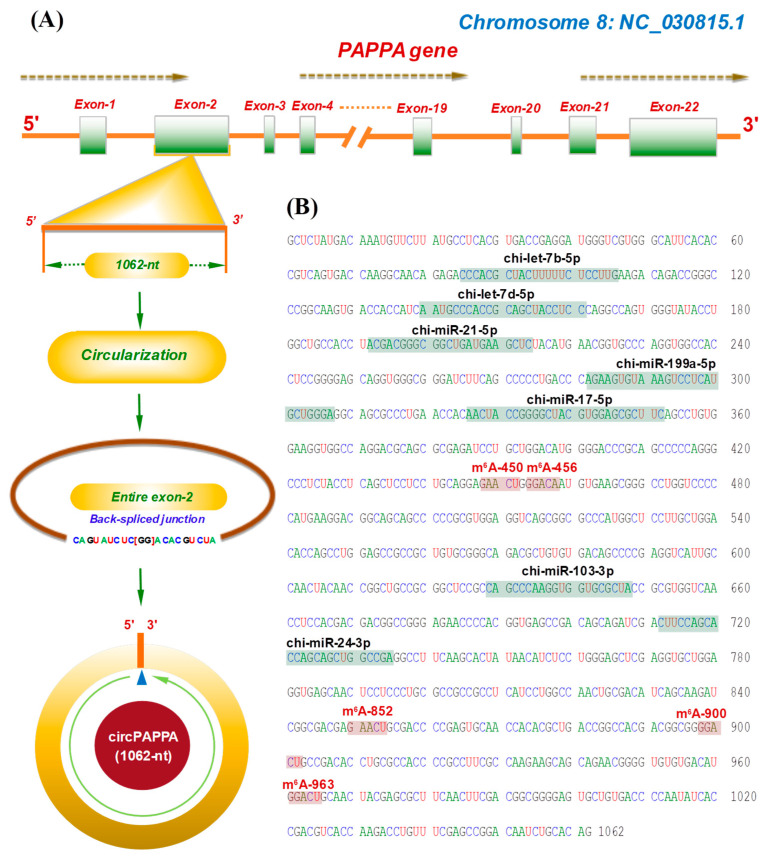
A diagram of circPAPPA host source in cashmere goats along with its sequence characteristics. (**A**) Structural feature diagram of the host gene of circPAPPA along with the reverse splicing size of 1062-nt. (**B**) Sequence display of circPAPPA that harbours seven possible binding sites of miRNAs, including chi-let7b-5p, chi-let7d-5p, chi-miR-21-5p, chi-miR-199a-5p, chi-miR-17-5p, chi-miR-103-3p, and chi-miR-24-3p. Also, five potential m^6^A modification sites were revealed within the circPAPPA molecule, including m^6^A-450, m^6^A-456, m^6^A-852, m^6^A-900, and m^6^A-963 with the motif of GAACU, GGACA, GAACU, GGACU, and GGACU, respectively.

CircPAPPA (also defined as chi_circ_2829 with a spliced length of 1062-nt) was previously identified from cashmere goat skin, where *PAPPA* had been determined as its host gene [17]. To further define its precise transcription region within the *PAPPA* gene, an alignment was performed by a linear sequence of circPAPPA against genome datasets of goat (*Capra hircus*). As shown in Figure 1A, the goat *PAPPA* gene (on chromosome 8) consists of 22 exons, which have been annotated in NCBI datasets (https://www.ncbi.nlm.nih.gov). CircPAPPA was spliced in reverse orientation by the entire exon 2 of the *PAPPA* gene (Figure 1A).

A preliminary bioinformatics analysis revealed five potential m^6^A modification sites within the circPAPPA molecule, including m^6^A-450, m^6^A-456, m^6^A-852, m^6^A-900, and m^6^A-963. Motif structures of these m^6^A modification sites are GAACU, GGACA, GAACU, GGACU, and GGACU, respectively (Figure 1B), which is fully consistent with the m^6^A motif structure previously reported in linear RNAs: RRACH (R: A/G and H: A/C/U) [25]. There were seven possible binding sites of miRNAs, including chi-let7b-5p, chi-let7d-5p, chi-miR-21-5p, chi-miR-199a-5p, chi-miR-17-5p, chi-miR-103-3p, and chi-miR-24-3p (Figure 1B). These results suggest that potential functional roles of circPAPPA in cashmere goat SHFs may be mediated by both the m^6^A modification sites and the miRNAs, as reported in a recent investigation [16].

### 3.2. Validation of circPAPPA m^6^A Sites and Its Subcellular Localization Along with Potential Binding Structure with Target miRNAs

**Figure 2:**
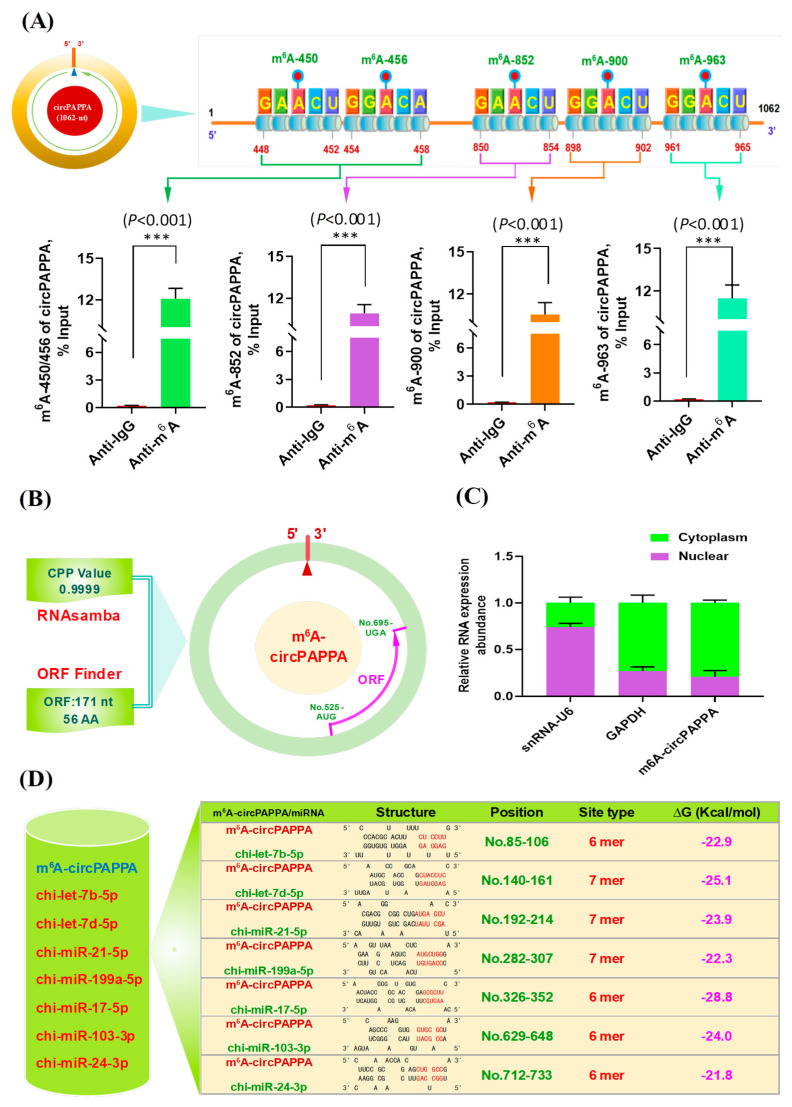
Validation of circPAPPA m^6^A modification sites and its subcellular localization, along with potential target miRNA prediction based on in silico analysis. (**A**) A diagram of potential m^6^A modification sites within circPAPPA along with their validation through Me-RIP technique followed by qPCR analysis. (**B**) Evaluation of circPAPPA coding potentiality along with a screening of potential ORFs. (**C**) Subcellular localization of circPAPPA in SHF stem cells of cashmere goats where the relative expression of *snRNA-U6* and *GAPDH* were also measured as internal controls of the nuclear and cytoplasm RNA, respectively. (**D**) Binding structure features of circPAPPA with its potential target miRNAs: chi-let7b-5p, chi-let7d-5p, chi-miR-21-5p, chi-miR-199a-5p, chi-miR-17-5p, chi-miR-103-3p and chi-miR-24-3p. The ∆G value was calculated using an online service program, RNAhybrid, under default settings (https://bibiserv.cebitec.uni-bielefeld.de/rnahybrid/, last access: 25 September 2024).

Three m^6^A modification sites (m^6^A-852, m^6^A-900, and m^6^A-963) were individually confirmed through the methylation immunoprecipitation (Me-RIP) technique followed by qPCR analysis (Figure 2A). However, we were unable to design respective primer pairs for m^6^A-450 and m^6^A-456 sites due to their rather close positional distance within the circPAPPA molecule. Alternatively, we designed a pair of primers (circPAPPA-m^6^A-450/456) that included these two possible m^6^A modification sites (m^6^A-450 and m^6^A-456) within their potential amplification region. The expected results were obtained in Me-RIP along with qPCR analysis (Figure 2A), but we still could not determine which one of the two sites (m^6^A-450 and m^6^A-456) is modified or whether both are modified by m^6^A methylation. We strongly recommend that further validation experiments be carried out on these two possible m^6^A modification sites (m^6^A-450 and m^6^A-456) via appropriate analytical assay and techniques. Interestingly, increasing lines of evidence indicate that the functional roles of m^6^A-modified circRNAs (m^6^A-circRNAs) are significantly mediated by their m^6^A modification sites [10,13]. We speculate that the biological functions of circPAPPA in SHF physiological processes of cashmere goats may be significantly regulated by the validated m^6^A sites, including m^6^A-450/m^6^A-456, m^6^A-852, m^6^A-900, and m^6^A-963.

On the other hand, circRNAs were initially considered a kind of non-coding RNA without the ability to encode proteins or peptides. However, an increasing number of studies have found that many circRNAs can encode biologically functional proteins or peptides [26]. We further evaluated the potential ability of circPAPPA to encode proteins or peptides. As a result, we found that circPAPPA contained one potential open reading frame (ORF) of 171-nt that could potentially encode a PAPPA-26aa peptide (Figure 2B). In fact, it has been reported that m^6^A modifications of circRNA play important roles in driving its effective initiation of translation in biological cells [27]. We strongly recommend further identification of the potential PAPPA-56aa peptide and verification of its biological functions, which may imply essential roles in SHF physiology and cashmere growth in cashmere goats.

As is well known, the subcellular localization of non-coding RNAs is essentially implicated in their biological significance [28]. We performed a detection of circPAPPA subcellular localization in the hair follicle stem cells of cashmere goats. CircPAPPA was expressed in both the nucleus and cytoplasm of the analyzed cells, with higher abundance in the cytoplasm (Figure 2C). In fact, it has been demonstrated that many circRNAs expressed in the cytoplasm can regulate the availability of miRNAs that bind with target mRNAs through competing endogenous RNA (ceRNA) pathway mechanisms [29]. We further analyzed the binding structure of circPAPPA with its predicted above-target miRNAs, including chi-let7b-5p, chi-let7d-5p, chi-miR-21-5p, chi-miR-199a-5p, chi-miR-17-5p, chi-miR-103-3p, and chi-miR-24-3p. As shown in Figure 2D, a fine base pairing structure can be formed between circPAPPA and its potential target miRNAs. Thus, it can be inferred that the circPAPPA function in cashmere goat SHFs might be achieved through the ceRNA mechanism mediated by the miRNAs, as reported in previous investigations [13,15].

### 3.3. CeRNA Regulatory Network of circPAPPA Along with Pathway Enrichment on Its miRNA Mediated Target Genes

It has been widely accepted that circRNAs can serve as miRNA sponges through competitively binding with miRNAs, thereby ultimately regulating the expression of target protein-coding genes [30]. In order to understand possible regulatory mechanisms of circPAPPA in cashmere goat SHFs, we established a ceRNA regulatory network of circPAPPA and the seven predicted target miRNAs along with their respective target protein-coding genes.

**Figure 3:**
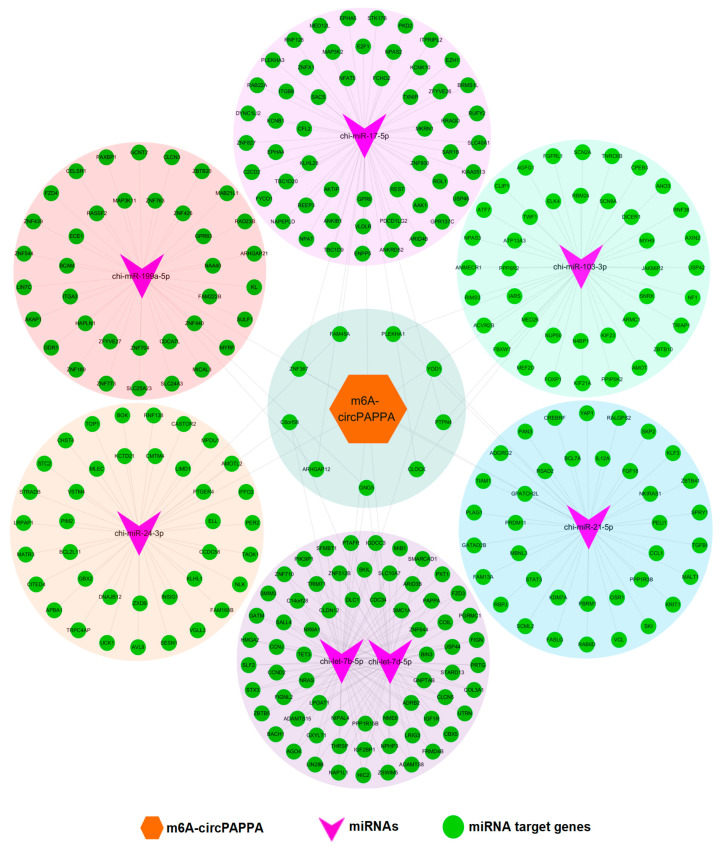
CeRNA regulatory network of cashmere goat circPAPPA that was constructed using the Cytoscape software (version 2.8.3). CircPAPPA was indicated by the brown hexagon. The miRNAs were indicated by purple swallowtail shapes. The potential target genes of miRNAs were indicated by green circles.

As shown in Figure 3, there is a complex regulatory relationship between circPAPPA and potential binding miRNAs along with their target protein-coding genes. Among the analyzed miRNAs, interestingly, four miRNAs were verified to exhibit significantly different expression in the skin during SHF cycles of cashmere goats, including chi-miR-199a-5p, chi-miR-17-5p, chi-miR-103-3p and chi-miR-24-3p [31]. Thus, it can be inferred that these miRNAs might be significantly implicated in the optimum balance of establishing gene expression in cashmere goat SHFs during SHF cycles, which may be important to maintain SHF physiological processes, including SHF regeneration and subsequent cashmere growth. These processes may be ultimately regulated by circPAPPA via ceRNA mechanism pathway as described by Shang and colleagues [32].

Some of the miRNA target genes in the ceRNA network have important biological significance in the regeneration and development of hair follicles. As an example, IGF1R acts on the transition from anagen to catagen in the hair follicle cycle partly via BMP4 activation [33]. It was also reported that KLF3, along with CNKSR2 and TNPO1, were associated with hair follicle development in sheep [34]. Herein, both *IGF1R* and *KLF3* were predicted as potential target genes of chi-let7-b/d-5p and chi-miR-21-5p, respectively (Figure 3). Also, *AXIN2* and *FOXP1* were revealed to be potential target genes of chi-miR-103-3p. In a previous investigation, AXIN2 was implicated in maintaining the quiescence of hair follicle stem cells, which may be achieved through autocrine Wnt/β-catenin signaling [35]. FOXP1 plays a role in regulating the proliferation of hair follicle stem cells during hair follicle cycles [36]. Based on these results, circPAPPA may be involved in several physiological processes of cashmere goat SHFs by modifying the expression level of target genes mediated by the above-predicted seven potential target miRNAs (Figure 3).

**Figure 4:**
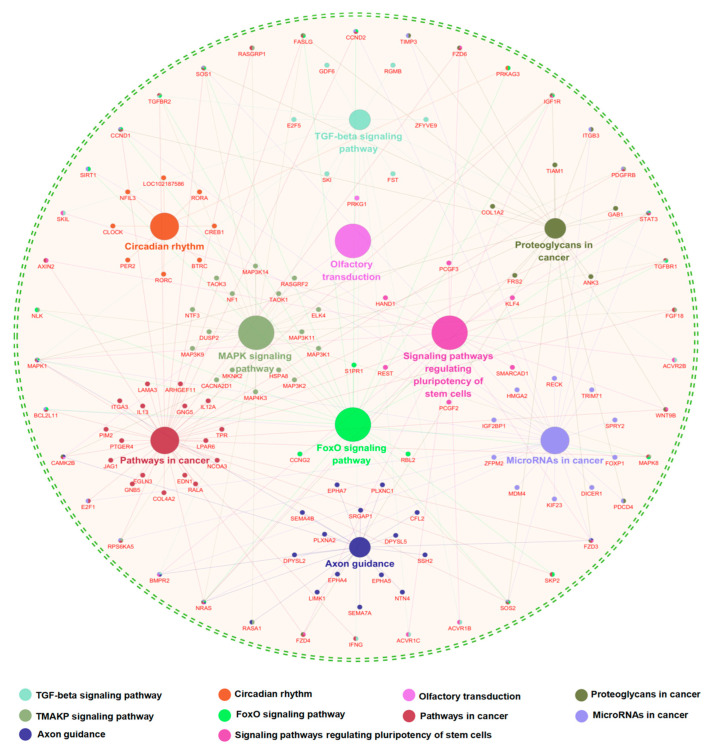
Signaling pathway enrichment of circPAPPA regulatory genes mediated by its potential target miRNAs. Enrichment of signaling pathways was performed by the CluePedia plugin embedded in Cytoscape software. Enriched results were presented as a network where each term of signaling pathways and its associated genes shared the same color.

To further explore the possible molecular mechanisms of circPAPPA functions in cashmere goat SHFs, a signaling pathway enrichment was conducted on its potential regulatory genes mediated by the predicted miRNAs. The analyzed genes were significantly enriched into several signaling pathways that play important roles in the development and growth of hair follicles, like Axon guidance, TGF-beta signaling pathway, Stem cell pluripotency regulatory pathway, and MAPK signaling pathway (Figure 4). It has been reported that the Axon guidance signal plays a role in the formation of hair follicles via driving rearrangement of large-scale cells [37]. It was demonstrated that TGF-beta signals could counteract BMP-mediated repression, thereby promoting the activation of hair follicle stem cells [38]. The stem cell pluripotency regulatory pathway has been proven to play a role in the differentiation of hair follicle stem cells into hair cells [39]. In addition, it is thought that the activation of the MAPK signaling pathway is, to a great extent, involved in the regulation of both the hair cycle and self-renewal of hair follicle stem cells [40]. Taken together, these findings provide meaningful insights into potential molecular mechanisms of circPAPPA in the SHF physiology of cashmere goats.

### 3.4. Regulatory Network and Pathway Enrichment of the Potential Regulatory Proteins of circPAPPA Molecule

**Figure 5:**
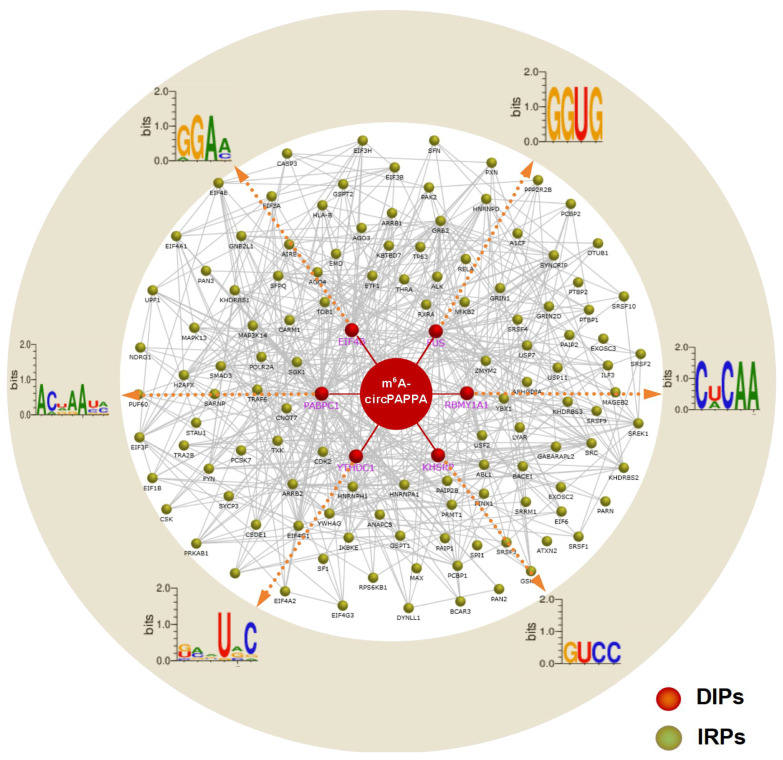
Regulatory network of m^6^A-circPAPPA in cashmere goats with its direct and indirect regulatory proteins. M^6^A-circPAPPA was indicated by a dark red circle. The proteins (DIPs) that directly interact with m^6^A-circPAPPA were indicated by dark red round balls where the binding motifs with each protein were provided in corresponding outer ring. The indirect regulatory proteins (IRPs) of m^6^A-circPAPPA mediated by DIPs were indicated by dark green round balls.

Increasing lines of evidence show that non-coding RNAs can exert their functions in various biological cells through binding with RNA-binding proteins [41]. We also explored potential RNA-binding proteins of circPAPPA and constructed a regulatory network of circPAPPA with its putative binding proteins. Six RNA-binding proteins were revealed to have potential interacting relationships with m^6^A-circPAPPA, including EIF4B, FUS, RBMY1A1, KHSRP, YTHDC1, and PABPC1 (Figure 5). Furthermore, there are further extensive regulatory relationships between each of them and additional proteins (Figure 5).

It is currently unknown whether these six RNA-binding proteins (EIF4B, FUS, RBMY1A1, KHSRP, YTHDC1, and PABPC1) play direct roles in the SHF physiology of cashmere goats. However, several of them have been proven to be involved in pivotal signaling pathways related to the development and growth of hair follicles. For example, FUS, PABPC1, and EIF4B were implicated in the MAPK [42], Nothch1 [43] and TGF-beta [44] signaling pathways, respectively. Additionally, YTHDC1 has been identified as a nuclear m^6^A modification reader [45]. In fact, it is thought that RNA-binding proteins can regulate multiple aspects of cellular physiology via binding motifs within circRNA molecules. However, the binding motifs of circRNAs can be hidden within their local conformation, thereby preventing the interactions of circRNA and RNA-binding proteins [46]. Although it is still unknown how m^6^A modification of circRNA mediates the interaction of circRNAs with RNA-binding proteins, we speculate that m^6^A modification of circPAPPA may alter its local conformation to promote the accessibility of binding motifs within circPAPPA molecules, which further facilitates the interactions of circPAPPA and the RNA-binding proteins as described in previous publications [13,15].

**Figure 6:**
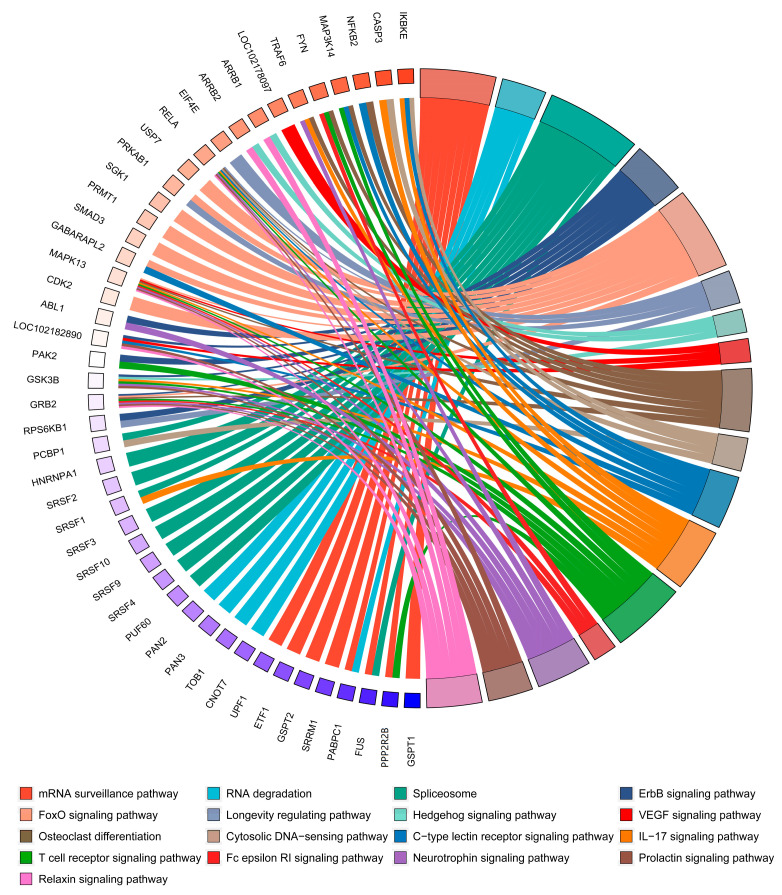
Signaling pathway enrichment of the potential regulatory proteins by the circPAPPA molecule. The enrichment analysis of signaling pathways was conducted using the CluePedia plugin embedded in Cytoscape software under default settings. The significantly enriched pathways were provided as a chordmap that was generated by the SRplot procedure [23].

Pathway enrichment was also carried out on the IRPs of circPAPPA based on in silico analysis. The analyzed proteins were significantly enriched into 17 signaling pathways (Figure 6). Of them, several pathways were confirmed to be closely associated with the development and growth of hair follicles. Fox example, it has been reported that VEGF signals can induce the proliferation of dermal papilla cells in human hair follicles [47], and Prolactin signaling is involved in regulating seasonal growth cycles of hair follicles in many mammals [48]. Hedgehog signaling was also revealed to reprogram fibroblasts of hair follicle niche into a highly activated status [5]. These findings suggest that the proteins analyzed in our study play significant roles in the SHF physiology of cashmere goats and may ultimately be regulated by circPAPPA molecules. Additionally, it is worth noting that some data in this study were obtained based on bioinformatics analysis. Therefore, it is hoped that future wet-lab experimental data will reveal the potential roles and functional mechanism of m^6^A-circPAPPA in the SHF physiology of cashmere goats.

### 3.5. Expression Pattern of m^6^A-circPAPPA and Its Potential Relationships with Methylation Within First Intron of PAPPA Gene in SHF Cycles of Cashmere Goats

**Figure 7:**
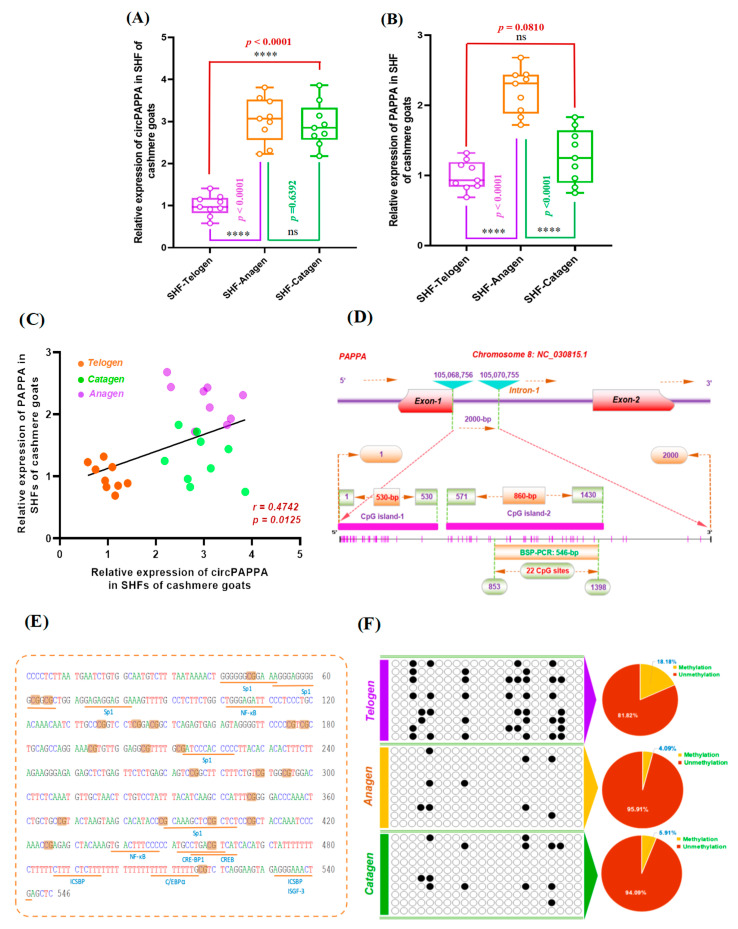
Expression features of circPAPPA in cashmere goat SHFs during hair follicle cycles and its potential relationships with the first intron methylation of the host gene PAPPA. (**A**) Relative expression of circPAPPA in cashmere goat SHFs at differential stages of hair follicle cycles. (**B**) Relative expression of the *PAPPA* gene in cashmere goat SHFs at differential stages of hair follicle cycles. (**C**) Expression correlation of circPAPPA and its host gene *PAPPA* in cashmere goat SHFs at differential stages of hair follicle cycles. (**D**) Prediction of CpG islands within the *PAPPA* gene’s first intron where the CpG sites were designated by pink vertical lines. The nucleotide positions were designated based on the *PAPPA* gene sequence in goat genome datasets: NC_030815.1 (Genome assembly ARS1.2, https://www.ncbi.nlm.nih.gov/datasets/genome/GCF_001704415.2). BSP stands for bisulfite sequencing PCR. (**E**) Prediction analysis of potential binding sites of transcription factors (underlined with yellow) in BSP analysis region within the *PAPPA* first intron of goats. The CpG sites were designated by yellow shadow regions. (**F**) Methylation analysis results of the first intron of the *PAPPA* gene in cashmere goat SHFs during differential stages of the hair follicle: telogen, anagen and telogen. The methylated and unmethylated CpG sites were designated by the filled black and unfilled white circles, respectively. The corresponding percentages of methylated CpG sites within the first intron of the *PAPPA* gene were presented by pie charts for each investigated stage of cashmere goat SHFs. The ‘ns’ represents no significant difference, and the ‘****’ indicates *p* < 0.0001.

It is widely believed that the expression of circRNAs exhibits highly spatiotemporal specificity during the developing phases of both tissues and organs, with their biogenesis accurately controlled by a series of regulatory factors [49]. CircPAPPA and its host gene (*PAPPA*) exhibited highly dynamic expression patterns in the SHFs of cashmere goats during hair follicle cycles [17], which were further verified in this investigation (Figure 7A,B). Although the expression trend of circPAPPA is not completely consistent with the linear RNA of its host gene (*PAPPA*), a significantly positive association has been revealed between them during the SHF cycle of cashmere goats with a correlation coefficient of 0.4742 (Figure 7C). A previous study showed that the first intron methylation of protein-coding genes is negatively associated with their expression level regardless of tissue or species [50]. Furthermore, there is a high degree of similarity in expression regulation between circRNAs and the linear RNAs of protein-coding genes [51]. Thus, we have reason to ask whether the first intron methylation of the *PAPPA* gene (i.e., the host gene of circPAPPA) may be involved in the dynamic expression pattern of circPAPPA in cashmere goat SHFs during telogen, anagen and catagen.

We further performed an investigation on the first intron methylation status of the *PAPPA* gene in cashmere goat SHFs during telogen, anagen and catagen. Two CpG islands, named CpG island-1 and CpG island-2, were identified within the first intron directly downstream to exon 1 of the *PAPPA* gene with a length of 530-nt and 860-nt, respectively (Figure 7D). A 546 bp fragment was amplified within CpG island-2 that spanned 22 CpG sites with several possible binding sites of transcription factors, such as Sp1, NF-kB, CRE-BP1, ICSBP and C/EBPα (Figure 7E). We detected the methylation degree of the amplified first intron region of the *PAPPA* gene in cashmere goat SHFs by pooling nine DNA samples into three groups: telogen, anagen, and catagen. The methylated ratios of 18.18%, 4.09% and 5.91% were revealed in the analyzed CpG sites corresponding to telogen, anagen and catagen, respectively (Figure 7F). Taken together with our above results, both anagen and categen have a higher circPAPPA expression in cashmere goat SHFs but have a lower methylation degree in the first intron region of the *PAPPA* gene in comparison to the telogen stage. Whereas telogen has lower expression of circPAPPA in cashmere goat SHFs, but has higher methylation levels in the analyzed first intron region of the *PAPPA* gene compared with both anagen and categen stages (Figure 7A,F). Thus, a higher degree of methylation within the first intron of the *PAPPA* gene may suppress circPAPPA biosynthesis in cashmere goat SHFs.

Considering the fact that circPAPPA is spliced in reverse orientation by the entire exon 2 of the *PAPPA* gene (Figure 1A), our results seem to support the previous findings that biosynthesis of exon-derived circRNA is closely associated with methylation at flanking introns [52]. However, it is worth noting that we only have detected the methylation status of the beginning region of the first intron of the *PAPPA* gene in cashmere goat SHFs (Figure 7D) and have not investigated methylation of the direct upstream and downstream regions of exon 2 within the *PAPPA* gene. Therefore, we can not draw a strong conclusion about whether the expression of circPAPPA is directly subjected to regulation by flanking introns methylation in cashmere goat SHFs. Also, we have not identified any variants of circPAPPA in cashmere goat SHFs. Therefore, it needs further exploration whether there are circPAPPA variants, including m^6^A-modifed and non-m^6^A-modified variants in cashmere goat SHFs. Currently, the mechanism by which epigenetic modifications regulate the expression of circRNAs in biological cells is unclear [20,52]. These findings suggest that the higher methylation level within the first intron of the *PAPPA* gene at telogen SHFs in cashmere goats is likely involved in the lower expression of circPAPPA during this phase. This may, at least in part, explain the dynamic expression pattern of circPAPPA in cashmere goat SHFs across anagen, catagen and telogen stages (Figure 7A,D). Overall, these results enhance our understanding of the epigenetic regulation of circPAPPA expression in cashmere goat SHFs.

## 4. Conclusions

The m^6^A-circPAPPA has been revealed to harbour at least four m^6^A modification sites in SHFs of cashmere goats, including m^6^A-450/456, m^6^A-852, m^6^A-900, and m^6^A-963. It exhibits higher expression in the cytoplasm of SHF stem cells of cashmere goats in comparison to the nucleus. The m^6^A-circPAPPA may play multiple functional roles in SHF development and cashmere growth in goats through the potential regulatory network mediated by miRNAs and RNA-binding proteins. The first intron methylation of the m^6^A-circPAPPA host gene (*PAPPA*) is most likely significantly involved in the dynamic expression of m^6^A-circPAPPA in cashmere goat SHFs.

## Figures and Tables

**Table 1:** Details of PCR primers that were utilized in this study, along with the corresponding amplicon size and annealing temperature.

Gene/Site Name	References	Sequence (5′-3′) ^a^	Primer Length (nt)	Amplicon Size (bp)	Annealing Temperature (°C)
m^6^A-circPAPPA (Divergent primers)	Yin et al., 2019 [17]	F: CACCAAGACCTGTTTCGAGC R: GGTCACGTGAGGCATAAGAAC	20 21	71	59
circPAPPA-m^6^A-450/456	The present study	F: CCCCCAGGGCCCTCTACC R: GGCTGCTGCCGTCCTTCA	18 18	89	58
circPAPPA-m^6^A-852	The present study	F: GCCGCCGCCTCATCCTGG R: CGTGGCCGGTCAGCGTGT	18 18	90	61
circPAPPA-m^6^A-900	The present study	F: CCCCGAGTGCAACCACAC R: CCCCGTTCTGCTGCTTCT	18 18	92	58
circPAPPA-m^6^A-963	The present study	F: AGAAGCAGCAGAACGGGG R: GGTGATATTGGGGTCACA	18 18	89	55
*GAPDH*	Yin et al., 2023 [15]	F: TGAACCACGAGAAGTATAACAACA R: GGTCATAAGTCCCTCCACGAT	24 21	125	53
*snRNA-U6*	Yin et al., 2020 [15]	F: CGCTTCGGCAGCACATATAC R: AAATATGGAACGCTTCACGA	20 20	Not available	55
*PAPPA* (BSP-primers)	NC_030815.1 in Genbank ^b^	F: TTTTTTTTAATGAATTTGTGGT R: AAACTCAATTTCCCTCTACTTCC	22 23	546	58

^a^ F: forward, R: reverse. ^b^ GenBank: https://www.ncbi.nlm.nih.gov (last access: 18 February 2024).

## Data Availability

The data presented in this study are not publicly available, and the data are available on request from the corresponding author.

## Abbreviations

The following abbreviations are used in this manuscript:

m^6^A	*N*^6^-methyladenosine
circRNA	Circular RNA
Me-RIP	Methylation immunoprecipitation
ceRNAs	Competing endogenous RNAs
ORF	Open reading frame
DIPs	Direct interaction proteins
IRPs	Indirect regulatory proteins
